# The Effect of Direct and Indirect Barbecue Cooking on Polycyclic Aromatic Hydrocarbon Formation and Beef Quality

**DOI:** 10.3390/foods12071374

**Published:** 2023-03-23

**Authors:** Gulsah Sumer, Fatih Oz

**Affiliations:** Department of Food Engineering, Agriculture Faculty, Ataturk University, Erzurum 25240, Turkey

**Keywords:** barbecue, direct cooking, indirect cooking, polycyclic aromatic hydrocarbons, lipid oxidation, beef

## Abstract

Herein, the effect of direct and indirect barbecue cooking processes, including different cooking degrees (medium and well done), on the formation of polycyclic aromatic hydrocarbons (PAHs) and on certain quality parameters (water content, cooking loss, pH, lipid oxidation) of beef meat was examined. While no significant effect (*p* > 0.05) of the cooking method was detected on the water content, cooking loss, ∑PAH4 [the sum of benzo[a]pyrene (BaP), benzo[a]anthracene (BaA), chrysen (Chry), and benzo[b]fluoranthene (BbF)], and ∑PAH8 [the sum of BaP, BaA, Chry, benzo[k]fluoranthene (BkF), dibenzo[a,h]anthracene (DahA), benzo[g,h,i]perylene (Bghip), and indeno [1,2,3-cd]pyrene (IncdP)] content, it was determined that it had a significant effect on pH (*p* < 0.05) and lipid oxidation (TBARS, *p* < 0.01). While the cooking degree did not have a significant effect (*p* > 0.05) on the TBARS value, it had a significant effect (*p* < 0.05) on the water content and pH value, and a very significant effect (*p* < 0.01) on the cooking loss. While BaA and BaP compounds were detected in all barbecued samples, the DahA compound could not be detected in any of the samples. Varying levels of BaA (up to 5.62 ng/g), Chry (up to 0.43 ng/g), BbF (LOD-..-LOQ), BkF (LOD-..-LOQ), BaP (up to 0.49 ng/g), BghiP (up to 0.82 ng/g), and IncdP (up to 4.99 ng/g) compounds were determined in the samples. While the ∑PAH4 contents varied between 0.71 and 6.35 ng/g, the ∑PAH8 contents varied between 1.12 and 11.34 ng/g. While the increase in cooking level did not affect the ∑PAH4 content, it caused a significant increase in the ∑PAH8 content. The highest BaP (0.49 ng/g), ∑PAH4 (6.35 ng/g), and ∑PAH8 (11.34 ng/g) contents were detected in the meat samples that were well cooked on the barbecue by the direct method. The results have proven that PAHs are formed at varying levels in both meat samples cooked on the barbecue by the direct method and the indirect method. On the other hand, it has been determined that even if 100 g of the meat with the highest BaP and ∑PAH4 content is eaten, the exposure amount remains far below the limit values specified for PAHs. However, paying close attention to the barbecue cooking process is still recommended.

## 1. Introduction

Nutrition can be defined as an action taken not only to create a feeling of satiety but also to protect health by providing the basic nutrients needed by the body and to increase the body’s resistance by providing a quality life. Foods contain many compounds that have been found to be beneficial to consumers. Animal-origin foods, which have an important place among our foods, especially meat and meat products, are one of the main sources of macro- and micro-compounds needed by the body [1,2,3,4,5]. It is stated that some of the micro-compounds present in meat are either absent entirely in plant-based foods or have very low bioavailability. This situation plays an important role in providing the body with nutrients (essential amino acids, essential fatty acids, vitamins, especially B group vitamins, and mineral substances, such as Fe and Zn) that benefit human health in many ways through meat consumption. In addition, meat consumption contributes to keeping the glycemic index at low levels, which causes many diseases (obesity, diabetes, cancer) with its high protein and low carbohydrate content [6,7].

Meat and meat products, except for products suitable for raw consumption, are usually consumed after heat treatment. The cooking process affects the color, taste–flavor, and textural properties of the meat. In addition, meat becomes easier to digest and almost sterile when cooked properly [8,9,10]. One of the widely used cooking methods of meat in the world is barbecue cooking. Barbecue cooking is based on dry cooking with heat sources, such as an oven and an electric or outdoor grill. In the barbecue cooking process, meats with a small amount of connective tissue are generally preferred. However, since the heat source radiates from one direction, the meat must be rotated during the process [11]. Barbecue cooking is frequently preferred by consumers due to the unique taste, aroma, and textural properties it gives to the product. The cooking process on the barbecue is essentially carried out in two ways: direct and indirect. In the direct cooking process, a wire grill is placed between the heat source and the food, and the smoke (incense) from the heat source comes into direct contact with the food. In the indirect cooking process, the heat source and the food are in different compartments, and the direct contact of the smoke with the food is separated, preventing the fat in the food from melting and dripping on the coal [12]. While the barbecue cooking process gives the products some unique important properties, it can also cause the formation of various heat treatment toxicants. One of these toxic substances are polycyclic aromatic hydrocarbons [13,14,15].

Polycyclic aromatic hydrocarbons (PAHs) are colorless, white, light yellow, and green in color, in solid form, have a slightly pleasant odor, and are pure compounds. In addition, due to the hydrophobic nature of PAHs, their solubility in water is very low and they have high lipophilic properties [16,17,18]. Different mechanisms have been proposed for PAH formation in foods, as follows: (1) pyrolysis of organic substances, such as fat, protein, and carbohydrates at high temperatures (500–900 °C); (2) encountering high temperatures as a result of fat droplets separated from the food cooked in coal fire falling on the burning coal; (3) formation of PAH compounds as a result of incomplete combustion of coal and their contamination on the food surface [12,18,19,20]. The physical and chemical properties of PAH compounds differ according to their molecular weights. PAHs with less than four benzene rings in their structure are classified as light PAHs, and PAHs with four or more benzene rings are classified as heavy PAHs, and most of them have been proven to have teratogenicity, carcinogenicity, and mutagenicity, which pose a great threat to human health [15,17]. In addition, while PAH compounds with a high molecular weight have low volatility and solubility properties, their volatility decreases further with the increase in the number of rings in the structure of PAHs. As the molecular weights of PAH compounds increase, the melting point and boiling point increase, and the vapor pressure decreases. Most PAH compounds have a melting point below 250 °C and a boiling point above 300 °C. According to some studies, it has been reported that PAHs with a molecular weight below 216 g/mol do not show carcinogenic properties, while PAHs with higher molecular weights have carcinogenic properties [17,18].

It is stated that approximately 660 different PAH compounds have been identified to date, and 16 PAHs have been selected as priority contaminants by the US-EPA because they are carcinogens and/or mutagens [21,22,23]. In addition, PAH compounds have been classified as human carcinogens (Group 1A) or probable carcinogens (Group 2A) by the International Agency for Research on Cancer (IARC) [24]. PAHs are stated to be the largest class of cancer-causing chemicals [25]. IARC defined benzo[a]pyrene (BaP), which is an indicator in terms of carcinogenic effect and formation among PAH compounds, as a human carcinogen. In line with epidemiological studies, it has been revealed that there is a strong relationship between the consumption of processed meat products and various types of cancer, such as colorectal, bowel, breast, prostate, and pancreatic [24,26,27]. As a result of the studies, it has been revealed that PAH compounds in meat and meat products are mostly formed by barbecue cooking or smoking processes [28]. The amount and type of PAH compounds vary depending on various factors, such as the type of meat, fat content, cooking conditions (method, temperature, time, equipment used, etc.), the type of fuel used, proximity to the heat source, and direct contact of food with fire [12,17,19,20,29].

It has been determined that direct contact with the heat source is a very important factor in PAH formation in meat and meat products, and carcinogenic PAHs cannot be detected if the melted fat does not drip onto the heat source during the cooking process on electric grills or other properly designed grills. In this area, research has emphasized that direct contact with the flame or grill should be avoided during the cooking of the meat at high temperatures [30,31]. There are already many studies in the literature investigating the effect of barbecue cooking on PAH formation in meat [13,14,22,26,32,33,34,35,36,37]; however, to the best of our knowledge, no study has been done to date that examines the effect of indirect barbecuing on the formation of PAH using a special type of barbecue frequently used in our country. Therefore, in the current study, the effect of the cooking process in barbecues, which were designed according to the direct and indirect methods, on various quality criteria (water, pH, TBARS, and cooking loss) and PAH formation of beef chops was investigated.

## 2. Materials and Methods

### 2.1. Raw Material

In the research, beef muscle (*M. longissimus dorsi*) obtained from the meat combine of Erzurum Meat and Milk Institution, Türkiye, was used as raw material. The meat brought to the laboratory under the cold chain was used after being sliced into the same sizes of 1.5 cm thickness.

### 2.2. Standards

The standard PAH mixture was purchased from Supelco (Bellefonte, PA, USA) and consisted of the following: 10 μg of naphthalene (Nap), acenaphthene (Ace), acenaphthylene (Ac), fluorene (Flu), phenanthrene (Phe), anthracene (AnT), fluoranthene (FluA), pyrene (Pyr), benzo[a]anthracene (BaA), chrysene (Chry), benzo[b]fluoranthene (BbF), benzo[k]fluoranthene (BkF), benzo[a]pyrene (BaP), dibenzo[a,h]anthracene (DahA), benzo[g,h,i]perylene (Bghip), and indeno[1,2,3-cd]pyrene (IncdP) in 1 mL of acetonitrile.

### 2.3. Methods

#### 2.3.1. Cooking Process

**Figure 1:**
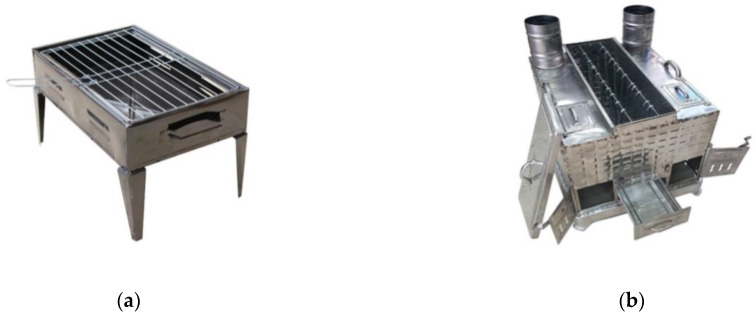
The photographs of the barbecues used in the current study. (**a**) Traditional barbecue for the direct method; (**b**) custom-made barbecue for the indirect method.

In the present study, the cooking process was carried out using two different designed barbecues (a traditional barbecue for the direct method and a custom-made barbecue for the indirect method) (Figure 1). These two types of barbecues have been frequently used in Türkiye. In both methods, the meat samples were cooked at two different cooking degrees (medium and well). In the cooking process, care was taken to ensure a homogeneous cooking process. For this purpose, for direct cooking, the samples cooked for a total of 6 min (3 + 3 min) were evaluated as medium-cooked samples, and the samples cooked for a total of 12 min (6 + 6 min) were evaluated as well-cooked samples. For the indirect cooking method, the samples cooked for a total of 11 min were considered medium-cooked samples, and the samples cooked for a total of 17 min were considered well-cooked samples. All meat samples were edible.

#### 2.3.2. Water Content

The water contents of the samples were determined based on the weight loss determined as a result of drying approximately 10 g meat samples in an oven (100 ± 2 °C) (approximately 18 h) until they reached a constant weight [38]. The formula used for the determination of water content is as follows:

$$Watercontent(\%)=\frac{\left(W1–W2\right)}{W3}\times 100$$

*W*1: First weighing (tare weight + sample weight before drying);

*W*2: Last weighing (tare weight + sample weight after drying);

*W*3: Sample weight before drying.

#### 2.3.3. Cooking Loss

The cooking loss of the meat samples was calculated based on the weight loss determined before the cooking process and after cooling to room temperature after the cooking process [39]. The formula used for the determination of cooking loss is as follows:

$$Cookingloss(\%)=\frac{\left(W4-W5\right)}{W4}\times 100$$

*W*4: Sample weight before cooking;

*W*5: Sample weight after cooking.

#### 2.3.4. pH Value

The pH value of the samples was determined using a pH meter after homogenizing 10 g of the sample with 100 mL of distilled water for about 1 min using ultra-turrax. Before using the pH meter, it was calibrated with appropriate buffer solutions (pH 4.0, 7.0, and 10.0) and then the reading process was performed [38].

#### 2.3.5. Determination of Lipid Oxidation Level (Thiobarbituric Acid Reactive Substances, TBARS)

The method given by Kılıç and Richards [40] was taken as a reference for the TBARS analysis. For this purpose, 2 g of meat samples were homogenized with 12 mL trichloroacetic acid (TCA) solution for 30 s using ultra-turrax (IKA Werk T 25, Staufen, Germany). Then, it was filtered through Whatman 1 filter paper and 3 mL of thiobarbituric acid (TBA) solution was added to 3 mL of filtrate. The mixture was kept in a 100 °C water bath for 40 min, then cooled in cold water and centrifuged at 2000 rpm for 5 min, and the absorbance values were determined against the blank sample at 530 nm in the spectrophotometer. TBARS values are given as mg malondialdehyde (MDA)/kg.

#### 2.3.6. Determination of Polycyclic Aromatic Hydrocarbon Content

The method given by Oz [29] was taken as a reference for the extraction method applied in the determination of PAH compounds. For this purpose, 15 mL of cold 1 M NaOH was added to 5 g meat sample and homogenized for 2 h. Then, 10 g Extrelut NT packaging material was added. The mixture was taken to the column and the PRS cartridge was connected and then washed with the PAH fraction dichloromethane. The collected fraction was then removed with nitrogen gas. The remaining part of the fraction was re-dissolved in 1 mL of n-hexane and placed on the column filled with 10 g of deactivated silica gel. Then, 25 mL n-hexane was passed through the column for pre-conditioning purposes. Subsequently, the column including the PAH fraction was washed with 60:40 (*v*/*v*) n-hexane and dichloromethane. The solvent was removed with nitrogen again and washed with 1 mL acetonitrile, then taken to the vials and a reading was made on the HPLC. PAHs were analyzed by applying gradient program with water and acetonitrile mobile phases using Hypersil™ Green PAH LC (150 mm × 2.1 mm, 3 μm) column in an HLPC device (Thermo Ultimates-3000, Waltham, MA, USA) with a fluorescent detector (FLD-3000). The mobile phase was deionized water as solvent A and acetonitrile as solvent B at a flow rate of 0.6 mL/min. The gradient program was as follows: 50% B, 0–22 min; 100% B, 22–24 min. The injection volume was 20 μL. The concentration of PAHs in the samples was calculated by a standard curve with different concentrations of standards. The quantification of PAHs was performed by an external calibration curve method. Coefficients of regression line (r^2^) for PAH standard curves were 0.9999 for BaA, 0.9999 for Chry, 0.9999 for BbF, 0.9999 for BkF, 0.9999 for BaP, 0.9999 for DahA, 0.9997 for Bghip, and 0.9991 for IncdP. Selected excitation/emission wavelengths were 270/390 nm for BaA and Chry, 260/430 nm for BbF, 290/410 nm for BkF, BaP, DahA and Bghip, and 290/470 nm for IncdP.

#### 2.3.7. Statistical Analysis

The current study was established according to the Randomized Complete Block Design and was carried out with three repetitions. The results were analyzed using the SPSS package program, and Duncan’s multiple comparison test (*p* < 0.05) was used to evaluate the differences between the mean values found to be significant [41].

## 3. Results and Discussion

### 3.1. Analysis Results of Raw Material

The water content, pH, and TBARS values of the meat samples used as raw material in the study were determined as 77.27 ± 0.05%, 5.69 ± 0.03, and 0.480 ± 0.020 mg MDA/kg, respectively. Similar results were obtained in the literature studies on beef [22,42].

### 3.2. Water Content Results of Cooked Meat Samples

The water contents of the meat samples cooked using direct and indirect cooking methods at different cooking degrees are presented in Table 1. As expected, the water content, which was determined as 77.27% in the raw samples, decreased as a result of cooking. The barbecuing reduces the water content of the samples due to the temperature reached, shrinkage of the myofibrillar proteins, and shrinkage of the perimysial connective tissue. Additionally, due to the high temperature applied during the cooking process, the water of the meat evaporates, which causes a decrease in the water content [43]. Although the water content (61.80%) of the samples cooked by the indirect cooking method was lower than that (62.26%) of the samples cooked by the direct cooking method, no difference (*p* > 0.05) was detected between them in terms of statistical evaluation. Generally considered, it would be expected that the process of cooking with the indirect cooking method would have shown a significant difference on the water content of the samples, resulting in lower water content. However, in the current study, the cooking times were kept different in order to cook the samples cooked by the direct and indirect methods at an equal level. Therefore, it is believed that the samples cooked by the indirect method are subjected to the cooking process for a longer time compared to the samples cooked by the direct method and that the exposure of both sides of the meat samples to heat during cooking affects this result. On the other hand, in a study investigating the effect of the use of wire and stone grills on the PAH content of meat samples in barbecue cooking [22], it was reported that the type of barbecue grill (wire and stone) had a significant effect on the water content of samples. The study reported the water content of meat samples cooked in wire barbecue as 56.98% and the water content of meat samples cooked in stone barbecue as 65.83%. In the current study, increasing the cooking degree of the samples caused a significant decrease in the water content. It is thought that the long cooking time affects this result.

### 3.3. Cooking Loss Results of Meat Samples

Cooking loss is one of the most important factors in terms of the quality and nutritional value of cooked meat. During cooking, there is a loss in the water-holding capacity of the meat. These losses, which are called cooking loss, depend on many factors (animal’s gender, age, muscle structure, cooking method and cooking time, etc.), and as a result of cooking, some physical, chemical, and microbiological changes, such as aroma formation, color, size, crispness, changes in fat content and protein fractions, mineral losses, and reduction of microbiological load, may be experienced in the product [44,45,46]. In the present study, the cooking loss values of the meat samples cooked using the direct and indirect cooking methods at different cooking degrees are also presented in Table 1. Although the cooking loss value (35.91%) of the samples cooked by the indirect cooking method was lower than that (36.39%) of the samples cooked by the direct cooking method, no significant difference (*p* > 0.05) was found between them. Oz and Yuzer [22] stated that the cooking loss values of the samples cooked on wire and stone grills were 42.61% and 34.45%, respectively, and they reported that the barbecue grill type (wire and stone) had no significant effect on the cooking loss of the samples. In the current research, increasing the cooking degree of the samples caused a significant increase (*p* < 0.01) in the cooking loss value. It was determined that the results showing the effect of cooking degree on cooking loss supported the results of water content obtained in the research. The lowest water content and the highest cooking loss values were determined in well-cooked meat samples. This result shows that in the present study, the main component removed from the meat as a result of the cooking process is the water of the meat. On the other hand, it is known that water is not the only component that moves away from meat in the losses that occur as a result of the cooking process; some components, such as water-soluble vitamins, minerals, and proteins, also move away from the meat along with meat water [43]. There are studies in the literature that cooking losses increase as the cooking temperature and/or degree of meat and meat products increase [2,47]. On the contrary, some studies have reported that the cooking degree has no effect on cooking loss [22]. Iwasaki et al. [48] reported that the cooking losses of rare, medium, well-done, and very well-done beef in Brazilian barbecue increased with the degree of cooking, and the cooking losses were 5, 31, 48, and 52%, respectively. In their study, Jinap et al. [49] determined the cooking loss as 25.45% in barbecued medium-cooked beef (satay) and 39.31% in well-done beef (satay), and reported that there was a statistical difference between the values. Similarly, Gu et al. [50] reported that the cooking loss value of barbecued beef increased from 31.3% to 55.0% with the increasing cooking degree (medium, good, and very good). Denaturation and coagulation of meat proteins during cooking reduces the water retention property of the meat, causing the meat to lose organoleptic properties and free water in the meat, as well as components, such as water-soluble vitamins, volatile and non-volatile aromatic substances, fat, and protein, which are broken down by the effect of heat, leaking out of the product [2,43,44,51].

### 3.4. pH Value Results of Cooked Meat Samples

The pH values of the meat samples cooked at different cooking degrees using direct and indirect cooking methods are also presented in Table 1. The pH value, which was determined as 5.69 in raw meat, increased in the meat samples cooked at different cooking degrees using direct and indirect cooking methods. After the cooking process, the release of bonds containing hydroxyl, sulfhydryl, and imidazole groups in the meat causes an increase in the pH value of the meat [52]. The pH value (5.95) of the samples cooked by the indirect cooking method was statistically (*p* < 0.05) lower than the pH value (5.99) of the samples cooked by the direct cooking method. On the other hand, it has been reported that there is no significant difference between the pH values of meat samples cooked on the barbecue using wire and stone grills [22]. The researchers reported the pH value of meat samples cooked on a wire barbecue as 5.71 and the pH value of meat samples cooked on a stone barbecue as 5.66. It is thought that the differences are due to factors such as raw materials, cooking conditions, etc. In the current study, the increase in the cooking degree of the samples caused a significant (*p* < 0.05) increase in the pH value. This result is thought to be influenced by the release of more alkaline compounds in meat samples exposed to high temperatures for a longer period of time under the conditions applied in the current research. Oz and Cakmak [53] and Oz et al. [54] determined that the pH values of the cooked beef meatball samples increased with the increase in cooking temperature. On the contrary, Oz and Yuzer [22] reported that the degree of cooking had no significant effect on the pH value of the samples in the barbecue cooking process.

### 3.5. TBARS Results of Meat Samples

Lipid oxidation is an important chemical reaction that causes rancidity in foods. Rancidity, in particular, occurs as a result of the reaction of unsaturated fatty acids with oxygen. The high amount of fat and degree of unsaturation of the product make the product more sensitive to lipid oxidation with the effects of processing, heat treatment, or storage. In addition, lipid oxidation affects the nutritional value, color, texture, taste, and aroma of the product [55,56,57]. The oxidation of fats in meat products can vary depending on many parameters, such as raw materials, additives, temperature, pH, catalysts, and time [58]. However, it is noted that one of the main causes of oxidation in meat and meat products is also heat treatment [59]. In the present study, TBARS values of the meat samples cooked by the direct and indirect cooking methods at different cooking degrees are also presented in Table 1. The TBARS value, which was determined as 0.480 mg MDA/kg in the raw meat, increased in meat samples cooked by the direct and indirect cooking methods at different cooking degrees. The reason for the increase in the TBARS value of meat samples as a result of heat treatment is explained as the lipid oxidation catalysis of iron released from myoglobin and hemoglobin compounds and the release of polyunsaturated fatty acids as a result of the destruction of the cell structure [60,61]. On the contrary, there are studies in the literature showing that the cooking process increases, decreases, or does not affect the TBARS value of meat depending on the cooking method [62,63,64,65,66]. In the current study, it was determined that the TBARS value (0.500 mg MDA/kg) of the samples cooked by the indirect cooking method was statistically lower (*p* < 0.05) than the TBARS value (0.603 mg MDA/kg) of the samples cooked by the direct cooking method. It is believed that this result is due to the better penetration of heat into the samples cooked by the direct method compared to the samples cooked by the indirect method and the decrease in the activation energy required for the reaction to take place due to heat exposure. A similar effect was observed in the pH values of the samples, and it was determined that the pH value of the samples cooked by the direct method was higher than that of the samples cooked by the indirect method, due to the release of alkaline compounds more. In the current study, increasing the cooking degree did not cause a significant effect (*p* > 0.05) on the TBARS value. This result shows that the activation energy required for lipid oxidation to occur is reached even in a medium cooking process. Similarly, Kilic [67] determined that the TBARS value of cooked beef samples after sealing at different levels varied between 0.905 and 1.692 mg MDA/kg, and the degree of sealing had no effect on the TBARS value. On the contrary, there are also studies in the literature reporting that cooking temperature does not have a significant effect on the TBARS value of beef meatballs prepared with different additives [47,66].

### 3.6. Polycyclic Aromatic Hydrocarbon Content Results of Cooked Meat Samples

The recovery values of PAHs varied between 49.03 and 92.57%. In addition, the limit of detection (LOD = 3) values ranged from 0.027 to 0.125 ng/g, while the limit of quantification (LOQ = 10) values ranged from 0.089 to 0.415 ng/g. It is seen that the LOD, LOQ, and recovery values determined in the current study are in agreement with the literature [68].

In the current study, a total of eight PAH compounds, benzo[a]anthracene (BaA), chrysen (Chry), benzo[b]fluoranthene (BbF), benzo[k]fluoranthene (BkF), benzo[a]pyrene (BaP), dibenzo[a,h]anthracene (DahA), benzo[g,h,i]perylene (Bghip), and indeno[1,2,3-cd]pyrene (IncdP), were investigated in the meat samples cooked at different cooking degrees using direct and indirect cooking methods. The individual PAH amounts of the meat samples cooked at different cooking degrees using direct and indirect cooking methods are presented in Table 2. While BaA and BaP compounds were determined in all of the samples analyzed, DahA compound was not detected in any of the samples. Various levels of BaA (up to 5.62 ng/g), Chry (up to 0.43 ng/g), BbF (<LOQ), BkF (<LOQ), BaP (up to 0.49 ng/g), BghiP (up to 0.82 ng/g), and IncdP (up to 4.99 ng/g) were detected in the samples depending on the cooking method and degree of cooking.

In the current study, BaA compound was detected in all meat samples cooked at different cooking degrees using direct and indirect cooking methods. BaA content of the meat samples cooked at medium level using direct cooking method was determined as 0.40 ng/g, while the BaA content of meat samples cooked at well level was determined as 5.62 ng/g. The BaA content of medium-cooked meat samples using the indirect cooking method was determined as 3.78 ng/g, while the BaA content of well-cooked meat samples was determined as 2.25 ng/g. In the meat samples cooked using the direct cooking method, an increase in the amount of BaA was observed depending on the degree of cooking, and a decrease was observed in the indirect cooking method. The highest BaA content (5.62 ng/g) was determined in well-cooked meat samples using the direct cooking method. Babaoğlu [69], in his study examining the effects of different meat types and different animal fats on the formation of PAH, determined that the BaA content in meat samples varied between 0.47 and 4.86 µg/kg. On the other hand, there are also studies in the literature in which BaA content could not be detected. As a matter of fact, Oz and Yüzer [22] reported that they could not detect BaA compound in any of the meat samples cooked at different levels on the barbecue using two different types of grills (wire and stone).

In the present study, while Chry compound could not be detected in the meat samples cooked at medium level using direct and indirect cooking methods, Chry compound was determined as 0.23 ng/g and 0.43 ng/g in the meat samples cooked well using direct and indirect cooking methods, respectively. Gosetti et al. [70] detected Chry compound as 0.152 ng/g in grilled beef tenderloin samples, while they detected it as 0.158 ng/g in beef tenderloin samples cooked on the grill with olive oil. Babaoğlu [69] reported that Chry content in meat samples varied between 0.56 and 5.60 µg/kg in his study examining the effects of different meat types and different animal fats on PAH formation. Aydın and Şahan [20], in their study investigating the effect of different cooking methods on PAH formation in beef, lamb, chicken, and turkey meat samples, reported that Chry compound was detected as 1.13 ng/g only in turkey meat samples. Oz and Yuzer [22] found that the Chry content of meat samples cooked in wire barbecue increased depending on the degree of cooking and varied between 0.12 and 0.14 ng/g. In addition, the researchers found a decrease in Chry content with increasing cooking degree in meat samples cooked on the stone barbecue and determined Chry compound at the level of 0.28 ng/g, 0.15 ng/g, and 0.12 ng/g in meat samples cooked medium, good, and very well on the stone barbecue, respectively.

In the current research, BbF compound could not be detected in the samples cooked at medium and well degree using indirect cooking method. On the other hand, BbF compound could not be detected in medium-cooked samples using the direct cooking method, while BbF compound was detected in well-cooked samples, but its amount could not be determined. Farhadian et al. [28] reported that the amount of BbF varied in the range of nd–13.8 ng/g in different samples of meat (veal, chicken, fish) cooked using different grill types (coal, gaseous) and oven. Babaoğlu et al. [14] determined that the BbF content varied between 0.50 and 5.37 µg/kg in the meat samples they analyzed in their research. Oz and Yuzer [22] reported that BbF content varied between nd–0.33 ng/g in meat samples cooked on a wire barbecue, and between nd–0.39 ng/g in meat samples cooked on a stone barbecue.

In this study, BkF compound could not be detected in the medium and well-cooked samples using the indirect cooking method. On the other hand, BkF compound could not be detected in medium cooked samples using the direct cooking method, while BkF compound was detected in well-cooked samples, but its amount could not be determined. Gosetti et al. [70] determined the BkF content as 1303 ng/g in the samples of beef tenderloin cooked on the grill and as 1351 ng/g in the samples of beef tenderloin cooked on the grill with olive oil. Onyango et al. [71] reported that the BkF content of beef samples cooked using local cooking methods varied between 0.143 and 0.202 ng/g. Oz and Yuzer [22] detected the BkF compound in meat samples cooked at different levels on the barbecue using two different grill types (wire and stone), only in meat samples cooked at medium level on a stone grill (0.90 ng/g). Babaoglu et al. [14], in their study examining the effect of using different animal fats on PAH formation in beef and lamb kokorec, reported that the BkF content of beef kokorec varied between nd–2.83 ng/g.

In the present study, BaP compound, which is described as an indicator of PAHs, was detected in all meat samples cooked at different cooking degrees using direct and indirect cooking methods. BaP content also increased depending on the degree of cooking in meat samples cooked by both methods. The BaP content of the meat samples cooked at medium level using the direct cooking method was determined as 0.31 ng/g, while the BaP content of the meat samples cooked at the well level was determined as 0.49 ng/g. The BaP content of the meat samples cooked at medium level using the indirect cooking method was determined as 0.27 ng/g, while the BaP content of meat samples cooked at well level was determined as 0.39 ng/g. Oz and Yüzer [22] reported that BaP content varied between nd-0.26 ng/g in meat samples cooked on a wire grill and between nd–0.29 ng/g in meat samples cooked on a stone grill. Aaslyng et al. [34] reported that the highest BaP content (24 µg/kg) was detected in beef samples among beef, pork, and chicken samples cooked in barbecue. Duedalh-Olesen et al. [26] reported that the average BaP content varied between 0 and 63 µg/kg in different meat samples (beef, pork, chicken, salmon, and lamb) cooked on barbecue. Oz [29] detected the BaP compound in all samples of meatballs produced using different animal fats and reported that the amount of BaP varied between 2.33 and 4.30 ng/g.

In the present study, DahA compound could not be detected in any of the meat samples cooked at different cooking degrees using direct and indirect cooking methods. Gosetti et al. [70] determined the DahA content as 0.434 ng/g in grilled beef steak samples and as 0.450 ng/g in beef steak cooked in olive oil. Babaoğlu et al. [14] reported that the DahA content of beef kokorec in which they prepared different animal fats varied between 0.46 and 3.25 ng/g. On the other hand, there are also studies in the literature showing that the DahA compound could not be detected. As a matter of fact, Oz and Yüzer [22] reported that they could not detect the DahA compound in meat samples cooked at different levels on the barbecue using two different types of grills (wire and stone). Similarly, Onopiuk et al. [12] were not able to detect DahA compound in their smoked and grilled meat products.

In the current study, BghiP compound could not be detected in any of the meat samples cooked at different cooking degrees using the direct cooking method. On the other hand, it was determined that the amount of BghiP compound detected in all meat samples cooked by the indirect cooking method increased in parallel with the increase in the degree of cooking. It was determined that the samples cooked with the indirect cooking method contained 0.51–0.82 ng/g of BghiP compound. Similarly, Oz and Yuzer [22] reported in their study that BghiP compound could not be detected in meat samples cooked in wire barbecue. The researchers reported that in meat samples cooked on a stone grill, the BghiP compound was determined only in medium-cooked meat samples (0.43 ng/g). Babaoglu et al. [14] reported that the content of BghiP in beef kokoreç samples, in which they produced different animal fats, varied between 1.80 and 3.66 ng/g.

In this study, IncdP compound could not be detected in the samples medium-cooked by the indirect cooking method, while IncdP compound was detected at the level of 0.48 ng/g in well-cooked samples. On the other hand, it was determined that the amount of IncdP compound detected in all meat samples cooked by the direct cooking method increased in parallel with the increase in cooking degree. It was determined that the samples cooked by the direct cooking method contained IncdP compound between 0.41 and 4.99 ng/g. Oz and Yuzer [22] reported that they could not detect the IncdP compound in meat samples cooked at different levels on the barbecue using two different types of grills (wire and stone). Onyango et al. [71] reported that the IncdP content of different meat samples cooked using different cooking methods varied between 0.097 and 0.253 ng/g.

It has been reported by the European Union Food Safety Authority (EFSA) that it would be better to use ∑PAH4, which expresses the sum of BaP, BaA, Chry, and BbF, and ∑PAH8, which expresses the sum of BaA, Chry, BbF, BkF, BaP, DahA, BghiP, and IncdP, instead of using only BaP compound to interpret PAH levels in foods [72]. Therefore, in the present study, the average of ∑PAH4 and ∑PAH8 contents of the meat samples cooked at different cooking degrees using direct and indirect cooking methods are calculated and also presented in Table 2. The ∑PAH4 contents of the meat samples cooked at medium and well levels by the direct cooking method were determined as 0.71 ng/g and 6.35 ng/g, respectively, while the ∑PAH8 contents were determined as 1.12 ng/g and 11.34 ng/g, respectively. The ∑PAH4 contents of the meat samples cooked at medium and well levels using the indirect cooking method were determined as 4.06 ng/g and 3.07 ng/g, respectively, ∑PAH8 contents were determined as 4.57 ng/g and 4.37 ng/g, respectively. While it was determined that the ∑PAH4 and ∑PAH8 contents of the samples cooked with the direct cooking method increased depending on the degree of cooking, it was observed that the ∑PAH4 and ∑PAH8 contents of the samples cooked with the indirect cooking method decreased as a result of the increase in the cooking degree. While Oz and Yuzer [22] could not determine the PAH compounds in the samples cooked at low and medium degrees using a wire grill, they determined the ∑PAH4 contents as 0.77 ng/g and 0.87 ng/g in the meat samples cooked well and very well, respectively. While the researchers could not determine the PAH compounds in the rare-cooked samples using a stone grill, they determined the ∑PAH4 contents as 1.30 ng/g, 0.92 ng/g, and 0.78 ng/g in the meat samples that they cooked medium, well and very well levels, respectively. The researchers reported that the ∑PAH4 and ∑PAH8 contents of the samples they barbecued using a wire grill were the same. On the other hand, they reported that the ∑PAH4 and ∑PAH8 contents of the meat samples that they cooked well and very well levels on the barbecue using a stone grill were the same, but the ∑PAH8 content was 2.63 ng/g in the samples that they cooked at medium level. Aaslyng et al. [34] stated in their study that among barbecued beef, pork, and chicken meat samples, the highest ∑PAH4 and ∑PAH8 contents were determined in beef samples and ∑PAH4 and ∑PAH8 contents varied between 0.4 and 65 μg/kg, and 18–867 μg/kg, respectively. Gorji et al. [73] reported that the ∑PAH4 contents of the meat samples they cooked in different barbecue types ranged between 1.87 and 6.91 and ∑PAH8 contents ranged between 2.39 and 7.73 ng/g. Aydın and Şahan [20], in their study investigating the effect of different cooking methods on PAH formation in beef, lamb, chicken, and turkey meat samples, determined PAH4 compounds only in barbecued meat samples.

The statistical comparison of the average of ∑PAH4 and ∑PAH8 contents of the meat samples cooked at different cooking degrees using direct and indirect cooking methods are given in Table 3. It was determined that the cooking method did not have a statistically significant effect on the ∑PAH4 and ∑PAH8 contents of the samples (*p* > 0.05). This shows that both ∑PAH4 and ∑PAH8 contents were formed at similar levels in both cooking methods. As it is known, in the direct cooking method of the barbecue cooking, it is possible that the fat drops that are removed from the meat due to the temperature reached during cooking, drip on the coal and form smoke, and this smoke reaches the meat again and creates more PAH compounds. On the other hand, in the indirect cooking method, the presence of coal and meat in different sections avoids this problem. Therefore, the samples cooked by the indirect cooking method would be expected to have lower levels of ∑PAH4 and ∑PAH8 content compared to samples cooked by the direct cooking method. However, in the current research, it is thought that the fact that the cooking process is started after the coal is completely converted into ember in the direct cooking method, the use of a relatively less fatty piece of meat (chop) as a material in the cooking process, and the application of heat from both surfaces of the meat in the indirect cooking method as opposed to the direct cooking method affect this result. Finally, in the present study, it was determined that there was no significant difference between the ∑PAH4 contents and ∑PAH8 contents of the samples cooked at different levels by the direct and indirect methods. As the cooking degree increased, both ∑PAH4 and ∑PAH8 contents of the samples increased, but this increase was only significant in ∑PAH8 content.

Oz [29] investigated the effect of the use of different animal fats on PAH formation in meatballs and reported that the ∑PAH4 content ranged from 8.41 to 15.48 ng/g. The researcher reported that the high level of PAH4 content was caused by the use of different animal fats. Duedalh-Olesen et al. [26], on the other hand, reported that the average total PAH content of different meat samples (beef, pork, chicken, salmon, and lamb) they cooked on the barbecue varied between 0.1 and 195 μg/kg.

Various PAH compounds are formed during the cooking processes, such as barbecue or grilling of meat and meat products, and this formation mainly occurs by the pyrolysis of the fat in the food as a result of contact with fire, or by the contamination of the meat surface by the smoke formed by the fat dripping into the flame [29,68]. It has been proven that in addition to the fat content in the food, the heat treatment time and the distance to the heat source are also important in the formation of PAH. For this reason, the type/shape of the grill and the distance of the heat source can be listed as the main factors affecting the formation of PAH in barbecued meat products [29,68,74]. On the other hand, in the study conducted by Min et al. [75], it was reported that cooking temperature is more effective than cooking time in the formation of PAHs. Adeyeye and Ashaolu [76] emphasized that PAH levels can be reduced by increasing the distance between the food and the heat source since PAHs cling to smoke particles and contaminate the food.

To sum up, it was determined that the individual PAH contents determined in the present study were generally compatible with the data in the literature. It is thought that some of the differences seen are due to differences in meat type, meat preparation techniques, cooking conditions, extraction and chromatographic analysis techniques, etc.

## 4. Conclusions

In the present study, it was determined that different levels of PAH compounds were formed in the meat samples cooked at different degrees using direct and indirect methods. It is observed that the results obtained in the current research are generally lower when compared with the data in the literature. Due to the carcinogenic and/or mutagenic effects of PAH compounds, their amounts have been restricted in various foodstuffs. The maximum presence limit in cooked meat and meat products is specified as 5 g/kg for BaP and 30 g/kg for ∑PAH4 [77]. In the present study, the highest BaP (0.49 ng/g), ∑PAH4 (6.35 ng/g), and ∑PAH8 (11.34 ng/g) contents were detected in the meat samples that were well cooked in the barbecue by the direct method. It has been determined that even if 100 g of the meat with the highest BaP and ∑PAH4 content is eaten, it remains far below the limit values specified for PAHs.

## Figures and Tables

**Table 1:** The water content, cooking loss, and pH value of the meat samples cooked using direct and indirect cooking methods at different cooking degrees (mean ± SD).

	n	Water (%)	Cooking Loss (%)	pH	TBARS (mg MDA/kg)
Cooking method					
Direct	6	62.26 ± 4.10a	36.39 ± 7.30a	5.99 ± 0.01a	0.603 ± 0.05a
Indirect	6	61.80 ± 3.74a	35.91 ± 6.54a	5.95 ± 0.07b	0.500 ± 0.03b
Sign.		ns	ns	*	**
Cooking degree					
Medium	6	64.61 ± 3.22a	30.78 ± 4.22b	5.95 ± 0.06b	0.539 ± 0.05a
Well-done	6	59.44 ± 2.13b	41.53 ± 3.01a	5.99 ± 0.02a	0.565 ± 0.09a
Sign.		*	**	*	ns

Sign.: Significance; Different letters (a and b) in the same column are significantly different (*p* < 0.05); SD: standard deviation; ns: not significant (*p* > 0.05); *: *p* < 0.05; **: *p* < 0.01.

**Table 2:** The individual PAH contents (ng/g) of the samples cooked using direct and indirect cooking methods at different cooking degrees (mean ± SD).

Cooking Method	n	Cooking Degree	BaA	Chry	BbF	BkF	BaP	DahA	BghiP	IncdP	∑PAH4	∑PAH8
Direct	3	Medium	0.40 ± 0.18	nd	nd	nd	0.31 ± 0.13	nd	nd	0.41 ± 0.03	0.71 ± 0.08	1.12 ± 0.07
	3	Well	5.62 ± 2.97	0.23 ± 0.15	nq	nq	0.49 ± 0.26	nd	nd	4.99 ± 3.29	6.35 ± 2.73	11.34 ± 3.59
Indirect	3	Medium	3.78 ± 2.06	nd	nd	nd	0.27 ± 0.07	nd	0.51 ± 0.06	nd	4.06 ± 2.13	4.57 ± 2.12
	3	Well	2.25 ± 1.76	0.43 ± 0.24	nd	nd	0.39 ± 0.25	nd	0.82 ± 0.66	0.48 ± 0.10	3.07 ± 1.78	4.37 ± 2.41

SD: standard deviation; nd: not detected (<LOD); nq: not quantified (LOD < … < LOQ).

**Table 3:** ∑PAH4 and ∑PAH8 contents of the meat samples cooked using direct and indirect cooking methods at different cooking degrees (mean ± SD).

	N	∑PAH4	∑PAH8
Cooking method			
Direct	6	3.53 ± 3.53a	6.23 ± 6.04a
Indirect	6	3.56 ± 1.83a	4.47 ± 2.04a
Sign.		ns	ns
Cooking degree			
Medium	6	2.39 ± 2.73a	2.84 ± 2.32b
Well-done	6	4.71 ± 2.73a	7.86 ± 4.70a
Sign.		ns	**

Sign.: Significance; Different letters (a and b) in the same column are significantly different (*p* < 0.05); SD: standard deviation; ns: not significant (*p* > 0.05); **: *p* < 0.01.

## Data Availability

The data that support the findings of this study are available from the authors upon reasonable request.

## References

[1] Oz F., Kaban G., Kaya M. (2010). Effects of cooking methods and levels on formation of heterocyclic aromatic amines in chicken and fish with Oasis extraction method. LWT-Food Sci. Technol..

[2] Öz F., Kızıl M., Çakmak İ., Aksu M.İ. (2015). The effect of direct addition of conjugated linoleic acid on the formation of heterocyclic aromatic amines in beef chops. J. Food Process. Preserv..

[3] Ledesma E., Rendueles M., Díaz M.J.F.C. (2016). Contamination of meat products during smoking by polycyclic aromatic hydrocarbons: Processes and prevention. Food Control.

[4] Unal K., Karakaya M., Oz F. (2018). The effects of different spices and fat types on the formation of heterocyclic aromatic amines in barbecued sucuk. J. Sci. Food Agric..

[5] Oz E., Oz F. (2022). Mutagenic and/or carcinogenic compounds in meat and meat products: Heterocyclic aromatic amines perspective. Theory Pract. Meat Process..

[6] Biesalski H.K. (2005). Meat as a component of a healthy diet—Are there any risks or benefits if meat is avoided in the diet?. Meat Sci..

[7] Haskaraca G., Demirok Soncu E., Kolsarıcı N., Öz F., Juneja V.K. (2017). Heterocyclic aromatic amines content in chicken burgers and chicken nuggets sold in fast food restaurants and effects of green tea extract and microwave thawing on their formation. J. Food Process. Preserv..

[8] Oz F. (2014). Effects of water extract of *Urtica dioica* L. on the quality of meatballs. J. Food Process. Preserv..

[9] Nuray M., Oz F. (2019). The effect of using different types and rates of onion-water extract in meatball production on the formation of heterocyclic aromatic amines. J. Sci. Food Agric..

[10] Cordeiro T., Viegas O., Silva M., Martins Z.E., Fernandes I., Ferreira I.M., Calhau C. (2020). Inhibitory effect of vinegars on the formation of polycyclic aromatic hydrocarbons in charcoal-grilled pork. Meat Sci..

[11] Rose M., Holland J., Dowding A., Petch S.R., White S., Fernandes A., Mortimer D. (2015). Investigation into the formation of PAHs in foods prepared in the home to determine the effects of frying, grilling, barbecuing, toasting and roasting. Food Chem. Toxicol..

[12] Onopiuk A., Kołodziejczak K., Marcinkowska-Lesiak M., Poltorak A. (2022). Determination of polycyclic aromatic hydrocarbons using different extraction methods and HPLC-FLD detection in smoked and grilled meat products. Food Chem..

[13] Chung S.Y., Yettella R.R., Kim J.S., Kwon K., Kim M.C., Min D.B. (2011). Effects of grilling and roasting on the levels of polycyclic aromatic hydrocarbons in beef and pork. Food Chem..

[14] Babaoglu A.S., Karakaya M., Öz F. (2017). Formation of polycyclic aromatic hydrocarbons in beef and lamb kokorec: Effects of different animal fats. Int. J. Food Prop..

[15] Sun Y., Wu S., Gong G. (2019). Trends of research on polycyclic aromatic hydrocarbons in food: A 20-year perspective from 1997 to 2017. Trends Food Sci. Technol..

[16] Alver E., Demirci A., Özcimder M. (2012). Polisiklik aromatik hidrokarbonlar ve sağlığa etkileri. Fen Bil. Enst. Derg..

[17] Günç Ergönül P., Kaya D. (2015). Polisiklik aromatik hidrokarbonlar ve gıdalarda önemi. Celal Bayar Üniversitesi Fen Bilim. Derg..

[18] Kılıç Ö., Dinçer E.A., Erbaş M. (2017). Gıdalarda polisiklik aromatik hidrokarbon bileşiklerinin bulunuşu ve sağlık üzerine etkileri. Gıda.

[19] Farhadian A., Jinap S., Faridah A., Zaidul I.S.M. (2012). Effects of marinating on the formation of polycyclic aromatic hydrocarbons (benzo [a] pyrene, benzo [b] fluoranthene and fluoranthene) in grilled beef meat. Food Control.

[20] Aydın Ö.Ş., Şahan Y. (2018). Bazı et türlerinde polisiklik aromatik hidrokarbon oluşumuna farklı pişirme yöntemlerinin etkisi. Akademik Gıda.

[21] Stołyhwo A., Sikorski Z.E. (2005). Polycyclic aromatic hydrocarbons in smoked fish—A critical review. Food Chem..

[22] Oz F., Yuzer M.O. (2016). The effects of cooking on wire and stone barbecue at different cooking levels on the formation of heterocyclic aromatic amines and polycyclic aromatic hydrocarbons in beef steak. Food Chem..

[23] Masuda M., Wang Q., Tokumura M., Miyake Y., Amagai T. (2019). Simultaneous determination of polycyclic aromatic hydrocarbons and their chlorinated derivatives in grilled foods. Ecotoxicol. Environ. Safe..

[24] Malarut J.A., Vangnai K. (2018). Influence of wood types on quality and carcinogenic polycyclic aromatic hydrocarbons (PAHs) of smoked sausages. Food Control.

[25] Chen J., Chen S. (2005). Removal of polycyclic aromatic hydrocarbons by low density polyethylene from liquid model and roasted meat. Food Chem..

[26] Duedahl-Olesen L., Aaslyng M., Meinert L., Christensen T., Jensen A.H., Binderup M.L. (2015). Polycyclic aromatic hydrocarbons (PAH) in Danish barbecued meat. Food Control.

[27] Kim M.J., Kim S., Choi S., Lee I., Moon M.K., Choi K., Park J. (2021). Association of exposure to polycyclic aromatic hydrocarbons and heavy metals with thyroid hormones in general adult population and potential mechanisms. Sci. Total Environ..

[28] Farhadian A., Jinap S., Abas F., Sakar Z.I. (2010). Determination of polycyclic aromatic hydrocarbons in grilled meat. Food Control.

[29] Oz E. (2021). The presence of polycyclic aromatic hydrocarbons and heterocyclic aromatic amines in barbecued meatballs formulated with different animal fats. Food Chem..

[30] Jaegerstad M., Skog K. (2005). Genotoxicity of heat-processed foods. Mutat. Rese/Fundam. Mol. Mech. Mutagen..

[31] Farhadian A., Jinap S., Hanifah H.N., Zaidul I.S. (2011). Effects of meat preheating and wrapping on the levels of polycyclic aromatic hydrocarbons in charcoal-grilled meat. Food Chem..

[32] Chen B.H., Lin Y.S. (1997). Formation of polycyclic aromatic hydrocarbons during processing of duck meat. J. Agric. Food Chem..

[33] Kazerouni N., Sinha R., Hsu C.H., Greenberg A., Rothman N. (2001). Analysis of 200 food items for benzo [a] pyrene and estimation of its intake in an epidemiologic study. Food and Chem. Toxicol..

[34] Aaslyng M.D., Duedahl-Olesen L., Jensen K., Meinert L. (2013). Content of heterocyclic amines and polycyclic aromatic hydrocarbons in pork, beef and chicken barbecued at home by Danish consumers. Meat Sci..

[35] Erhunmwunse N.O., Ainerua M.O., Ogboghodo I.B., Ekene B. (2016). Effects of barbecuing on the levels of polycyclic aromatic hydrocarbons in fish (Pseudotolitus Elongatus and Clarias Gariepinus). J. Nat. Sci. Res..

[36] Kafouris D., Koukkidou A., Christou E., Hadjigeorgiou M., Yiannopoulos S. (2020). Determination of polycyclic aromatic hydrocarbons in traditionally smoked meat products and charcoal grilled meat in Cyprus. Meat Sci..

[37] Bostan S. (2021). Farklı Pişirme Yöntemlerinin Akçaabat Köftesinde Polisiklik Aromatik Hidrokarbon (PAH) Oluşumuna Etkisi. Master’s Thesis.

[38] Gökalp H.Y., Kaya M., Tülek Y., Zorba Ö. (2010). Et ve Ürünlerinde Kalite Kontrolü ve Laboratuvar Uygulama Klavuzu. (V. Baskı).

[39] Öz F., Kızıl M. (2013). Determination of heterocyclic aromatic amines in cooked commercial frozen meat products by ultrafast liquid chromatography. Food Anal. Methods.

[40] Kılıç B., Richards M. (2003). Lipid oxidation in poultry döner kebab: Pro-oxidative and anti-oxidative factors. J. Food Sci..

[41] Yıldız N., Bircan H. (1991). Uygulamalı İstatistik.

[42] Fencioglu H., Oz E., Turhan S., Proestos C., Oz F. (2022). The Effects of the marination process with different vinegar varieties on various quality criteria and heterocyclic aromatic amine formation in beef steak. Foods.

[43] Sánchez del Pulgar J., Gázquez A., Ruiz-Carrascal J. (2012). Physico—Chemical, textural and structural characteristics of sous—Vide cooked pork cheeks as affected by vacuum, cooking temperature, and cooking time. Meat Sci..

[44] Babür T.E., Gürbüz Ü. (2015). Geleneksel pişirme yöntemlerinin et kalitesine etkileri. J. Tour. Gastron. Stud..

[45] Şireli H.D. (2018). Karkaslarda et kalitesinin belirlenmesinde kullanılan geleneksel yöntemler ve yeni teknikler. Dicle Üniversitesi Fen Bilim. Enstitüsü Derg..

[46] Sanwo K.A., Adegoke A.V., Akinola O.S., Njoku C.P., Okolo S.O., Oladipo N.A., Oladejo A.S. (2019). Meat quality characteristics of improved indigenous chickens (FUNAAB-ALPHA) fed turmeric (*Curcuma longa*) or clove (*Syzygium aromaticum*) as feed additives. J. Agric. Sci. Environ..

[47] Kiliç S., Öz E., Öz F. (2021). Effect of turmeric on the reduction of heterocyclic aromatic amines and quality of chicken meatballs. Food Control.

[48] Iwasaki M., Kataoka H., Ishihara J., Takachi R., Hamada G.S., Sharma S., Tsugane S. (2010). Heterocyclic amines content of meat and fish cooked by Brazilian methods. J. Food Compos. Anal..

[49] Jinap S., Mohd-Mokhtar M.S., Farhadian A., Hasnol N.D.S., Jaafar S.N., Hajeb P. (2013). Effects of varying degrees of doneness on the formation of heterocyclic aromatic amines in chicken and beef satay. Meat Sci..

[50] Gu Y.S., Kim I.S., Park J.H., Lee S.H., Park D.C., Yeum D.M., Kım S.B. (2001). Effects of seasoning and heating device on mutagenicity and heterocyclic amines in cooked beef. Biosci. Biotechnol. Biochem..

[51] Khan I.A., Liu D., Yao M., Memon A., Huang J., Huanga M. (2019). Inhibitory effect of Chrysanthemum morifolium flower extract on the formation of heterocyclic amines in goat meat patties cooked by various cooking methods and temperatures. Meat Sci..

[52] Girard P.J. (1992). Cooking. Technology of Meat and Meat Products.

[53] Öz F., Çakmak İ.H. (2016). The effects of conjugated linoleic acid usage in meatball production on the formation of heterocyclic aromatic amines. LWT-Food Sci. Technol..

[54] Öz F., Kızıl M., Zaman A., Turhan S. (2016). The effects of direct addition of low and medium molecular weight chitosan on the formation of heterocyclic aromatic amines in beef chop. LWT-Food Sci. Technol..

[55] Aşçioğlu Ç. (2013). Farklı Pişirme Yöntemlerinin Sığır Bonfilelerinin (*Longissimus dorsi*) Besinsel ve Kalite Özellikleri Üzerine Etkisi. Master’s Thesis.

[56] Estévez M., Purslow P.P. (2017). What’s New İn Meat Oxidation. New Aspects of Meat Quality.

[57] Sabuncular G., Akbulut G., Yaman M. (2021). Ette Lipit oksidasyonu ve etkileyen faktörler. Avrupa Bilim Teknoloji Dergisi.

[58] Visessanguan W., Benjakul S., Riebroy S., Yarchai M., Wanaporn Tapingkae W. (2006). Changes in lipid composition and fatty acid profile of nham, a thai fermented pork sausage, during fermentation. Food Chem..

[59] Lepper-Blilie A.N., Berg E.P., Buchanan D.S., Keller W.L., Maddock-Carlin K.R., Berg P.T. (2014). Effectiveness of oxygen barrier oven bags in low temperature cooking on reduction of warmed– over flavor in beef roasts. Meat Sci..

[60] Ramirez M.R., Morcuende D., Estevez M., Lopez R.C. (2005). Fatty acid profiles of intramuscular fat from pork loin chops fried in different culinary fats following refrigerated storage. Food Chem..

[61] Rojas M.C., Brewer M.S. (2007). Effect of natural antioxidants on oxidative stability of cooked, refrigerated beef and pork. J. Food Sci..

[62] Alfaia C.M., Alves S.P., Lopes A.F., Fernandes M.J., Costa A.S., Fontes C.M., Prates J.A. (2010). Effect of cooking methods on fatty acids, conjugated isomers of linoleic acid and nutritional quality of beef intramuscular fat. Meat Sci..

[63] Juárez M., Failla S., Ficco A., Peña F., Avilés C., Polvillo O. (2010). Buffalo meat composition as affected by different cooking methods. Food Bioprod. Process..

[64] Zikirov E. (2014). Sous-Vide Pişirme Yönteminin Sığır Etinde Heterosiklik Aromatik Amin Oluşumu ve Bazı Kalitatif Kriterler Üzerine Etkisi. Master’s Thesis.

[65] Korkmaz A., Oz F. (2020). Effect of the use of dry breadcrumb in meatball production on the formation of heterocyclic aromatic amines. Br. Food J..

[66] Uzun I., Oz F. (2021). Effect of basil use in meatball production on heterocyclic aromatic amine formation. J. Food Sci. Technol..

[67] Kılıç S. (2021). Mühürleme işleminin etin Tekstür, Protein Profili ve Heterosiklik Aromatik amin Oluşumu Üzerine Etkisi. Master’s Thesis.

[68] Oz E. (2020). Effects of smoke flavoring using different wood chips and barbecuing on the formation of polycyclic aromatic hydrocarbons and heterocyclic aromatic amines in salmon fillets. PLoS ONE.

[69] Babaoğlu A.S. (2015). Dana ve Kuzu Kokoreçlerinde Polisiklik Aromatik Hidrokarbonların (PAH) Oluşum Düzeyi Üzerine Farklı Hayvansal Yağların Etkisi. Master’s Thesis.

[70] Gosetti F., Chiuminatto U., Mazzucco E., Robotti E., Calabrese G., Gennaro M.C., Marengo E. (2011). Simultaneous determination of thirteen polycyclic aromatic hydrocarbons and twelve aldehydes in cooked food by an automated on-line solid phase extraction Ultra High Performance Liquid Chromatography Tandem Mass Spectrometry. J. Chromatogr. A.

[71] Onyango A.A., Lalah J.O., Wandiga S.O. (2012). The effect of local cooking methods on polycyclic aromatic hydrocarbons (PAHs) contents in beef, goat meat, and pork as potential sources of human exposure in Kisumu City, Kenya. Polycyclic Aromat. Compd..

[72] European Food Safety Authority (EFSA) (2008). Polycyclic aromatic hydrocarbons in food-scientific opinion of the panel on contaminants in the food chain. EFSA J..

[73] Gorji M.E.H., Ahmadkhaniha R., Moazzen M., Yunesian M., Azari A., Rastkari N. (2016). Polycyclic aromatic hydrocarbons in Iranian Kebabs. Food Control.

[74] Zhu Z., Xu Y., Huang T., Yu Y., Bassey A.P., Huang M. (2022). The contamination, formation, determination and control of polycyclic aromatic hydrocarbons in meat products. Food Control.

[75] Min S., Patra J.K., Shin H.S. (2018). Factors influencing inhibition of eight polycyclic aromatic hydrocarbons in heated meat model system. Food Chem..

[76] Adeyeye S.A.O., Ashaolu T.J. (2020). Polycyclic aromatic hydrocarbons formation and mitigation in meat and meat products. Polycyclic Aromat. Compd..

[77] European Commission E.C. (2011). Commission Regulation (EU) No 835/2011 of 19 August 2011 amending Regulation (EC) No 1881/2006 as regards maximum levels for polycyclic aromatic hydrocarbons in foodstuffs. Off. J. Eur. Union.

